# Microbial Composition and Genes for Key Metabolic Attributes in the Gut Digesta of Sea Urchins *Lytechinus variegatus* and *Strongylocentrotus purpuratus* Using Shotgun Metagenomics

**DOI:** 10.3390/cimb43020070

**Published:** 2021-08-26

**Authors:** Joseph A. Hakim, George B. H. Green, Stephen A. Watts, Michael R. Crowley, Casey D. Morrow, Asim K. Bej

**Affiliations:** 1 Department of Biology, The University of Alabama at Birmingham, 1300 University Blvd., Birmingham, AL 35294, USA; joe21@uab.edu (J.A.H.); gg6255@uab.edu (G.B.H.G.); sawatts@uab.edu (S.A.W.); 2 Department of Genetics, Heflin Center Genomics Core, School of Medicine, The University of Alabama at Birmingham, 705 South 20th Street, Birmingham, AL 35294, USA; mcrowley@uab.edu; 3 Department of Cell, Developmental and Integrative Biology, The University of Alabama at Birmingham, 1918 University Blvd., Birmingham, AL 35294, USA

**Keywords:** echinoderm, gut microbiome, high-throughput sequencing, MG-RAST, KEGG, RefSeq, bioinformatics

## Abstract

This paper describes the microbial community composition and genes for key metabolic genes, particularly the nitrogen fixation of the mucous-enveloped gut digesta of green (*Lytechinus variegatus*) and purple (*Strongylocentrotus purpuratus*) sea urchins by using the shotgun metagenomics approach. Both green and purple urchins showed high relative abundances of Gammaproteobacteria at 30% and 60%, respectively. However, Alphaproteobacteria in the green urchins had higher relative abundances (20%) than the purple urchins (2%). At the genus level, *Vibrio* was dominant in both green (~9%) and purple (~10%) urchins, whereas *Psychromonas* was prevalent only in purple urchins (~24%). An enrichment of *Roseobacter* and *Ruegeria* was found in the green urchins, whereas purple urchins revealed a higher abundance of *Shewanella*, *Photobacterium*, and Bacteroides (*q*-value < 0.01). Analysis of key metabolic genes at the KEGG-Level-2 categories revealed genes for amino acids (~20%), nucleotides (~5%), cofactors and vitamins (~6%), energy (~5%), carbohydrates (~13%) metabolisms, and an abundance of genes for assimilatory nitrogen reduction pathway in both urchins. Overall, the results from this study revealed the differences in the microbial community and genes designated for the metabolic processes in the nutrient-rich sea urchin gut digesta, suggesting their likely importance to the host and their environment.

## 1. Introduction

The intertidal and nearshore marine ecosystems of North America harbor a diverse community of invertebrates, vertebrates, and microorganisms, with primary producers such as drift and benthic macroalgae and kelp forests and seagrass meadows that constitute a dynamic aquatic food web [1,2,3,4]. These ecosystems are enriched with inorganic and organic nutrients that are produced and utilized by the resident organisms. Among invertebrates, the sea urchins found in the nearshore coastal waters worldwide play a crucial role in the energy flow and nutrient cycling at various trophic levels [5]. In North America, the green sea urchin, *Lytechinus variegatus* (order Temnopleuroida, family Toxopneustidae), is generally found along the South Eastern Coast and into the Gulf of Mexico [6]. In contrast, the purple sea urchin, *Strongylocentrotus purpuratus* (order Echinoida, family Strongylocentrotidae), inhabits the U.S. Pacific coast from Alaska to Baja Mexico [7]. Although the sea urchins are omnivores, they mostly graze upon algae, kelp, seagrass, and decomposed materials [6,7,8,9,10,11]. The grazing activity by increased densities of sea urchins often severely limits seagrass and macroalgal biomass, resulting in a barren and depauperate ecosystem [12,13]. However, the grazing enables the sea urchins to metabolize and transform the ingested seagrass and macroalgal biomass into rich organic nutrients. Thus, despite their potentially damaging effect, sea urchins also play an essential ecological role in structuring the communities in their habitats [5,12,14,15,16,17]. 

Both green and purple urchins possess a deuterostome gut system in a relatively straightforward model [18]. The efficacy of the digestive process of the carbohydrate-rich seagrass and macroalgal biomass by sea urchins has been of interest due to their unique anatomical organization and the digestive enzymes described to be largely absent in the gut lumen [19]. Normally, the ingested foods are masticated by the Aristotle’s Lantern apparatus, which then enters the pharynx and esophagus, where a thick mucus layer is produced by specialized mucous-producing cells in the gut tissue envelops them. These gut digesta are physically separated from the luminal surface of the gut tissue and will remain intact throughout their passage and upon egestion [19]. Additionally, recent studies reported the enrichment of distinct microbial communities associated with the gut tissue of both the purple [20] and the green urchins. Previous studies described the crucial role of these microbial communities in the digestion and metabolism of the ingested seagrass and algae [19,21,22,23,24,25]. However, sea urchins are remarkably inefficient in assimilating these nutrients from the digesta [26].

As a result, a large quantity of mucous-enveloped high-energy egesta consisting of residual nutrients and microbiota is released in the ecosystem. These gut egesta are an important source of nutrients to marine organisms such as fish, crustaceans, shellfish, and other echinoderms [15,27,28,29]. The nutritional benefit of the green urchin egesta has also been shown to enhance the growth and the taste quality of shrimp *Litopenaeus vannamei* as compared to laboratory-formulated feed alone [14]. One important aspect of these nutrient transitions is nitrogen assimilation into amino acids and nucleotides, which are essential macromolecules for all living organisms [30]. Although various inorganic and organic nitrogen forms exist in the marine environment, nearly 80% of the global nitrogen budget exists in its unreactive diatomic form [31]. However, the metabolic productivity of primary producers depends on the availability of sufficient amounts of fixed nitrogen such as ammonia [32,33]. Moreover, assimilated organic nitrogen such as amino acids is crucial for marine heterotrophic animals to fulfill their nutritional requirements [34]. Thus, it has been suggested that the bacterial community in the sea urchin gut digesta play a vital role in the metabolism of nucleotides and amino acids through nitrogen fixation, which upon egestion provides essential nutrient to marine organisms [5,22,24,32,35].

The objective of this study was to use shotgun metagenomics to determine the taxonomic composition and metabolic profile of the microbial communities in the mucous-encapsulated gut digesta of the green and purple urchins using shotgun metagenomics. Moreover, these gut digesta samples were collected immediately following gut tissue dissection and gentle rinsing with appropriate sterile techniques to maintain the in situ microbial community. The study elaborates the signature genes designated for the carbohydrate, amino acid, and nucleotide metabolisms, emphasizing the nitrogen cycle. 

## 2. Materials and Methods

### 2.1. Microbial Metacommunity DNA for High-Throughput Sequencing

The collection of the green sea urchins, *L. variegatus*, from the Saint Joseph Bay Aquatic Preserve of the Gulf of Mexico Coast of Florida (Florida Coast) and the purple sea urchins, *S. purpuratus*, from Cape Argo of the Pacific Coast of Oregon (Oregon Coast) was performed as part of the previous investigations by our laboratory (Supplementary Figure S1) [20,25]. Briefly, adult green sea urchins (*n* = 3) were collected from within 1 m^2^ of each other at the Saint Joseph Bay Aquatic Preserve, Florida (29.80° N 85.36° W). Coastal water conditions were measured as 20 ± 2 °C, with a pH of 7.8 ± 0.2 and salinity of 28 ± 1 parts per thousand (ppt.). Adult purple sea urchins (*n =* 3) were collected from the same intertidal pool at Cape Arago, Oregon (43.3039° N 124.4014° W), and the water conditions were measured as 13.1 °C pH of 7.7 and salinity of 30.19 ppt. To collect the gut digesta, a radial incision was made around Aristotle’s Lantern mastication apparatus using sterile instruments, and the gut tissue was removed. The gut tissue was rinsed with autoclaved sterile phosphate-buffered saline water (1× PBS, pH 7.4) (Fisher Scientific, Hampton, NH, USA). The voided gut contents (gut digesta) were subsequently collected from each green (*n* = 3) and purple (*n* = 3) sea urchin and flash-frozen in liquid nitrogen until used. 

The metacommunity DNA from each gut digesta sample was purified using the Fecal DNA isolation kit (Zymo Research, Irvine, CA, USA; catalog no. D6010). For the green sea urchins, the purified metacommunity DNA of the biological replicate gut digesta (*n* = 3) samples were pooled into one representative DNA sample, which was subsequently aliquoted as two technical replicate DNA samples (*n* = 2) for shotgun sequencing. This procedure was similarly performed for the purple sea urchin. The purified DNA of replicates gut digesta (*n* = 3) samples was pooled and aliquoted to create two technical replicate DNA samples (*n* = 2) for sequencing. The technical replicate DNA samples for each sea urchin were prepared in duplicate to provide a repeated sequencing result for the same DNA samples and were thus subjected to shotgun sequencing in two different flow-cell lanes on an Illumina HiSeq 2500 sequencing platform at the UAB Heflin Center for Genomic Science (http://www.uab.edu/hcgs/ (accessed on 18 March 2018), using the paired-end (2 × 100) protocol described elsewhere [36]. The number of raw sequences generated by the Illumina HiSeq HTS has been described in Table 1.

### 2.2. MG-RAST Quality Checking and Sequence Read Processing

Initial sequence read quality was assessed using FastQC (v0.11.2) [37], and host DNA was filtered from the raw sequence data using Bowtie2 (v2.3.4.3) [38] against the most recent *Lytechinus variegatus* (assembly Lvar_2.2) and *Strongylocentrotus purpuratus* (assembly Spur_4.2) genome assembly from the National Center for Biotechnology Information (NCBI). Host-filtered paired-end sequences were then uploaded to Metagenomic Rapid Annotations using Subsystems Technology (MG-RAST; v4.0.3) [39] and processed using the default MG-RAST pipeline. Briefly, low-quality sequences were trimmed using the Dynamic Trim tool from SolexaQA [40], with the lowest PHRED quality score selected at 15, and sequences were trimmed if five nucleotides at most fell below this threshold. 

Using the MG-RAST (v4.0.3) analysis pipeline, initial sequence statistics were computed using DRISEE [41] and jellyfish [42], followed by adapter trimming through skewer [43]. Sequences were then filtered using the fastq-mcf tool [44] based on a minimum sequence length of 50 and a 10 nucleotides window size for trimming. RNA feature identification was performed using SortMeRNA [45], to determine those sequencing with an identity cut-off of 70% and an E-value of 0.1 to rRNA genes from a reduced M5RNA database [46] as implemented through MG-RAST. RNA clustering was performed using CD-HIT-EST [47] at a 97% threshold, and an RNA similarity search was performed using blat [48], followed by gene calling using FragGeneScan with protein-coding regions overlapping with rRNA genes masked from the downstream analysis. Amino acid sequence clustering was then performed at a 90% similarity through CD-HIT [47], and a protein similarity search was conducted through blat [48]. Abundance profiles per sample were then generated using the multiple taxonomic and gene databases through the MG-RAST automated pipeline (github.com/MG-RAST/pipeline) (accessed on 29 May 2018). 

For this analysis, taxonomic classifications were assigned using the NCBI Reference Sequence (RefSeq) Database [49]. Functional categories were assigned through the Kyoto Encyclopedia of Genes and Genomes (KEGG) Orthology (KO) Database [50,51], with the minimum sequence length set at 15 nucleotides at an E-value of 10^−5^ for a sequence match. The taxonomic count data at the highest resolution (species where possible) of domains were listed in table format (Supplementary Table S1). Rarefaction curve analysis was also performed on this data to show the taxonomic count against sequence depth (data not shown). The host-filtered sequence data were also checked using rarefaction analysis and domain Bacteria was extracted and subsampled to the minimum value across all samples (RefSeq = 646,758). The KO data were also subsampled to the minimum count value (KO = 206,021) [52]. 

### 2.3. Taxonomic Distribution 

The rarefied taxonomic relative abundance was visualized and plotted using Microsoft Excel software (Microsoft, Seattle, WA, USA). First, a relative abundance stacked column bar graph was generated to show the distribution of phyla (class for Proteobacteria) across all samples, and entries represented at <1% abundant across all samples were listed as “Other”. This relative abundance information at the phylum-level was also used to create a heatmap table using the conditional formatting option in Microsoft Excel Software. Colors were selected as “red” for less abundant, “yellow” for intermediate abundance, and “green” for high abundance. The relative abundance distribution at the genus level was also plotted as stacked column bar format, showing the top 50 taxa with the remaining taxa listed as “Other”. These data were also used for extended error bar analysis in Statistical Analysis of Metagenomic Profiles (STAMP; v2.1.3). For each urchin gut digesta, technical replicates comprising the annotated sequence data generated by the duplicate shotgun metagenomic sequencing were grouped, normalized, and a two-sided Welch’s *t*-test [53] was performed with *p*-values corrected using the Benjamini–Hochberg False Discovery Rate (FDR) approach [54]. The resultant *q*-values were set at <0.01 to indicate a significant differential abundance of genera between gut digesta groups.

### 2.4. Alpha and Beta Diversity of Taxa

Shannon [55] and Simpson [56] alpha diversity measures were performed at the most resolvable Operational Taxonomic Unit (OTU) level (species where possible) using the “alpha_diversity.py” command through QIIME (v1.9.1). This analysis was performed both before and after the filtration of those OTUs occurring only once across all samples, or singletons. Beta diversity was performed through heatmap analysis at the genus level. To do this, a heatmap was constructed in R (v3.3.2) using the heatmap.2 function from gplots (v3.0.1) package [57]. The sample dendrograms were constructed using the Vegan (v2.5-3) package according to the Bray–Curtis metric [58], and taxa represented at <1% were filtered out. The RColorBrewer package [59] was used to select the color palette at blue for more abundant and sky blue for least abundant, and a trace line was plotted (black bar lines) to show the percentage distribution.

### 2.5. Functional Analysis through KEGG Orthology 

Functional analysis was performed using the rarefied KEGG Orthology (KO) functional categories across all samples. First, the minimum subsampled KO count data were collapsed into their respective KEGG-Level-2 hierarchical category and plotted as relative abundance bar graphs using Microsoft Excel software (Microsoft, Seattle, WA, USA). The heightened categories (>1%) were shown, and the remaining low-abundant KEGG-Level-2 categories were listed as “Other”. Each included KEGG-Level-2 category was then ranked by average relative abundance within its higher KEGG-Level-1 category of metabolism, genetic information, environmental, and cellular processing. The KEGG-Level-1 category of metabolism was further analyzed to show the preferential abundance of KEGG-Level-3 KEGG function map Ids derived from the KEGG Pathway Database between the green and purple urchin gut digesta samples. To do this, the KEGG map Ids were grouped according to technical replicate and rendered as a scatter plot based on their relative proportion in the KEGG-Level-1 category of metabolism using STAMP (v2.1.3) analytical software [60]. Moreover, the metabolic pathways related to the KEGG-Level-3 nitrogen metabolism pathway were further investigated using KEGG Mapper [61] at the highest level of functional resolution (KO) and reconstructed as it relates to amino acid metabolism. The sequence counts assigned to each KO category in the pathway were listed in bar graphs through Microsoft Excel software (Microsoft, Seattle, WA, USA), and included alongside the direction of the indicated reactions. 

## 3. Results

### 3.1. Sequence Statistics 

The total number of raw sequences subjected to host DNA removal, followed by the quality-checked sequences from *L. variegatus* and *S. purpuratus* used for downstream bioinformatics analysis, are listed in Table 1. The taxonomic assignments from the quality-checked sequences and subsequent KO functional categories from both the green and purple urchin samples from a single pooled DNA sample of *L. variegatus* or *S. purpuratus* are also listed in Table 1. 

### 3.2. Taxonomic Distribution of the Microbial Community 

**Figure 1:**
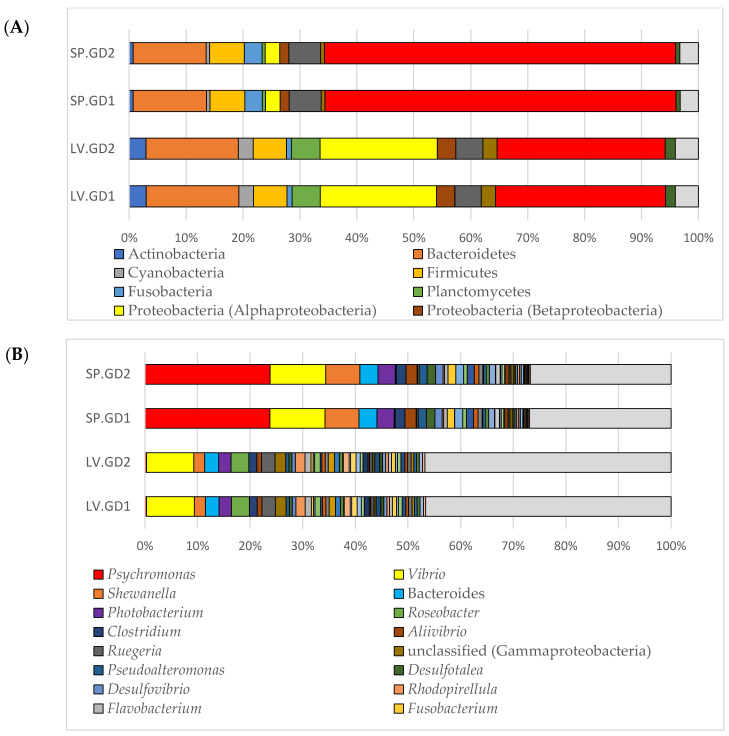
Relative abundance stacked column bar graphs illustrating the taxa distribution assigned to domain Bacteria through RefSeq as implemented in MG-RAST (v4.0.3). (**A**) The phylum level (class for Proteobacteria) distribution is shown, and phyla with an average abundance of <1% were listed as “Other”. (**B**) The relative abundance of the top 50 genera across all samples was plotted. The low represented taxa were categorized as “Other”. Bar graphs generated using Microsoft Excel software (Microsoft, Seattle, WA, USA), and samples are indicated as follows: LV.GD = green sea urchin *Lytechinus variegatus gut digesta*; SP.GD = purple sea urchin *Strongylocentrotus purpuratus* gut digesta.

For relative abundance distribution at the phylum (class for Proteobacteria) level, the green urchin digesta samples showed elevated Gammaproteobacteria (~30%) and Alphaproteobacteria (~20%) (Figure 1A). Comparatively, the purple urchin digesta samples showed Gammaproteobacteria to be most abundant at ~60%, whereas Alphaproteobacteria was only represented at ~2%. Of the commonly found phyla across both sea urchin digesta samples, the green urchins showed a slightly higher relative abundance of Bacteroidetes, Cyanobacteria, Actinobacteria, Planctomycetes, Verrucomicrobia, Beta-, and Epsilonproteobacteria, and the purple urchins had a slightly heightened Firmicutes, Fusobacteria, and Deltaproteobacteria (Figure 1A; Table 2). 

At the genus level, both the green and purple urchins gut digesta showed a heightened distribution of *Vibrio* (Figure 1B). However, the purple urchins showed uniquely heightened enrichment of *Psychromonas*. Of the commonly observed genera between the two groups, extended error bar analysis indicated *Roseobacter* and *Ruegeria* as significantly heightened in the green urchins compared to the purple urchins (*q*-value < 0.01) (Supplementary Figure S2).

Conversely, the purple urchin digesta showed *Shewanella*, *Photobacterium*, and Bacteroides to be significantly enriched compared to the green urchins (*q*-value < 0.01). The two-group Welch’s *t*-test results for each of the top 50 genera, including the *p*-values, Benjamini–Hochberg FDR-corrected *q*-values, and effect size differences, have been elaborated in Supplementary Table S1.

### 3.3. Alpha and Beta Diversity

**Figure 2:**
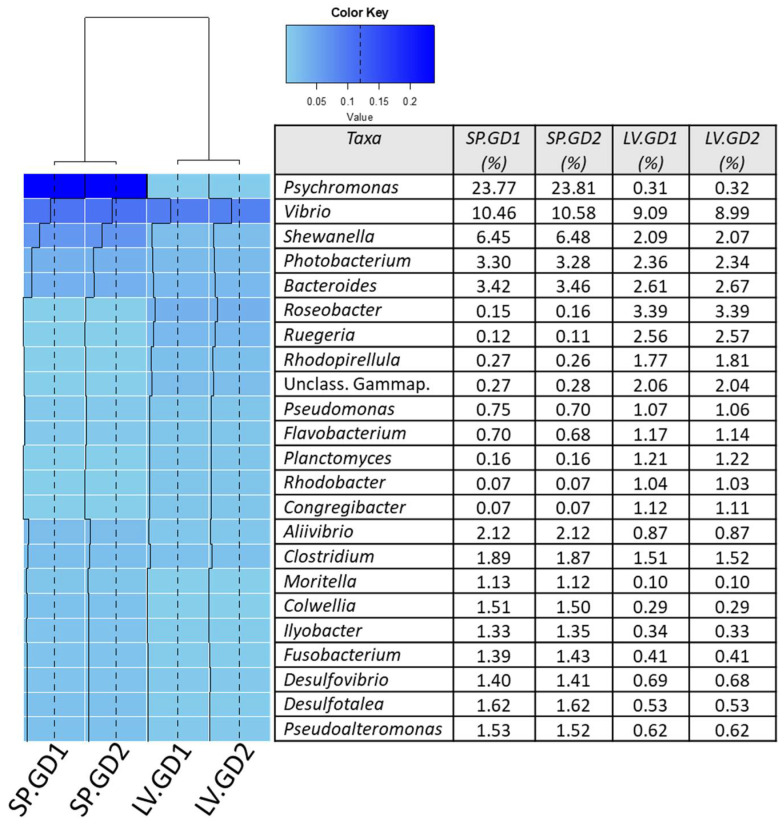
Heatmap analysis of the genera comprising domain Bacteria assigned through RefSeq using MG-RAST (v4.0.3). The analysis was performed using R (v3.3.2) with the heatmap.2 function from the gplots (v3.0.1) package. The sample dendrogram was constructed based on the Bray–Curtis similarity value through Vegan (v2.4.3), and the gradient of relative abundance was illustrated using RColorBrewer package as “blue” for more abundant and “sky blue” for less abundant. The trace lines (black) were generated to further elaborate on the relative abundance of taxa. Samples are indicated as follows: LV.GD = green sea urchin *Lytechinus variegatus* gut digesta; SP.GD = purple sea urchin *Strongylocentrotus purpuratus* gut digesta.

Alpha diversity of the samples in this study showed the green urchin digesta to have a higher Shannon and Simpson diversity than the purple urchin digesta samples (Table 1). The microbiota of both the LV.GD samples showed a Shannon of 8.99 and Simpson of 0.996, whereas the SP.GD showed a Shannon of 7.47 and Simpson of 0.965, with no variation between technical replicates. The removal of singletons showed no detectable difference in the alpha diversity across all samples. Additionally, alpha diversity performed on bacteria yielded a Shannon of 9.10 and Simpson of 0.996 for the LV.GD samples, and a Shannon 7.59 and Simpson of 0.967 for the SP.GD samples. Beta diversity demonstrated the technical replicates to cluster within a 97% Bray–Curtis similarity, and the beta diversity of the digesta across all samples was shown to be >60%, as shown through dendrogram cluster analysis (Figure 2). Heatmap analysis further elaborated the beta diversity and the contribution of heightened genera (>1%) to the observed variation of sample types (Figure 2).

### 3.4. Functional Categories Using the KEGG-Level-1 and Level-2 Orthology

**Figure 3:**
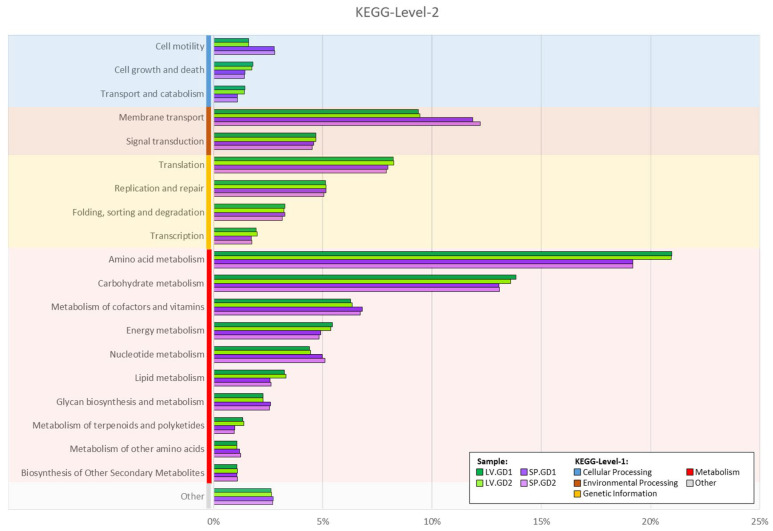
Relative abundance bar graphs were generated for each sample based on the KEGG-Level-2 categories determined through KEGG Orthology (KO) data following the MG-RAST (v4.0.3) workflow. KEGG-Level-2 categories were binned into their respective KEGG-Level-1 broad hierarchical functional category and ranked in decreasing order from top to bottom based on their average abundance. Each KEGG-Level-1 category was color-coded as follows: cellular processing = blue; environmental processing = brown; genetic information = orange; metabolism = red; and “other” = gray. The bar graphs for each sample have been color-coded and indicated as follows: SP.GD = purple sea urchin *Strongylocentrotus purpuratus* gut digesta; SP.GD1 = purple and SP.GD2 = light purple; LV.GD = green sea urchin *Lytechinus variegatus* gut digesta; LV.GD1 = green and LV.GD2 = light green.

The relative distribution of genes assigned showed the KEGG-Level-1 category of metabolism (global overview of carbohydrate, lipid, amino acids, energy, co-factor and vitamins, nucleotides, secondary metabolites, and terpenoid/polyketides, chemical structure transformation) to be the most heightened category, represented at approximately 60% for the green and 58% for the purple urchin samples. This was followed by genetic information processing (green = ~18% and purple = ~18%), and environmental information processing (green = ~14% and purple = ~16%). From the heightened KEGG-Level-1 category of metabolism, the KEGG-Level-2 categories showed amino acid and carbohydrate metabolism most prevalent in green and purple urchin digesta (Figure 3). This was followed by the metabolism of cofactors, vitamins, nucleic acid, and lipids. 

**Figure 4:**
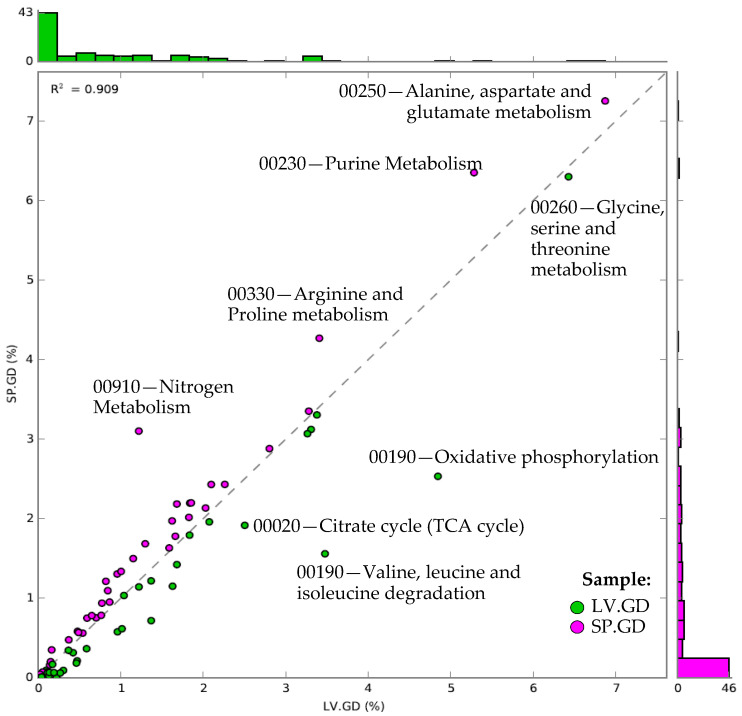
Relative abundance scatter plot analysis of the KEGG map Ids derived from the KEGG-Level-1 category of “metabolism”. KEGG map Id data was retrieved from the KEGG-Level-3 functional categories from MG-RAST (v4.0.3) and was uploaded into STAMP (v2.1.3). Technical replicates were grouped, and count data were normalized as the relative proportion of each KEGG map Id per group from the KEGG-Level-1 category of metabolism. The X-axis and Y-axis show each KEGG map Id relative abundance per group, including the histograms to show the number of functional entries falling at the specified abundance on the scatter plot. Those KEGG map Ids that were noticeably enriched in one group were indicated by their KEGG pathway name and Id number. The regression statistic (R^2^) was also shown in the plot. Sample groups and color codes are indicated as LV.GD = green sea urchin *Lytechinus variegatus* gut digesta (green); SP.GD = purple sea urchin *Strongylocentrotus purpuratus* gut digesta (purple).

The analysis of metabolic functions within the KEGG-Level-2 categories showed the most prevalent amino acid metabolism (Figure 4). Furthermore, the KEGG-Level-3 pathway assigned to alanine, aspartate, and glutamate (KEGG map Id: 00250) was prevalent in both the green and purple urchin digesta (Figure 4). However, when compared between the two urchin digesta, the purple urchins were higher than green urchins. In addition, glycine, serine, and threonine metabolism (KEGG map Id: 00260) were also demonstrated at greater abundance in the green urchins than purple urchins. Other heightened amino acid metabolism categories included arginine and proline (KEGG map Id: 00330) that favored the purple urchins, whereas valine, leucine, and isoleucine metabolism and oxidative phosphorylation (KEGG map Id: 00190) were favored in the green urchins. Categories involved in energy metabolism were also observed, showing nitrogen metabolism (KEGG map Id: 00910) more enriched in the purple urchins than the green urchins. The citrate cycle (KEGG map Id: 00020) and oxidative phosphorylation were enriched compared to the purple urchins in green urchins. 

### 3.5. Genes for the Assimilatory Reduction and Nitrogen Fixation into Ammonia 

**Figure 5:**
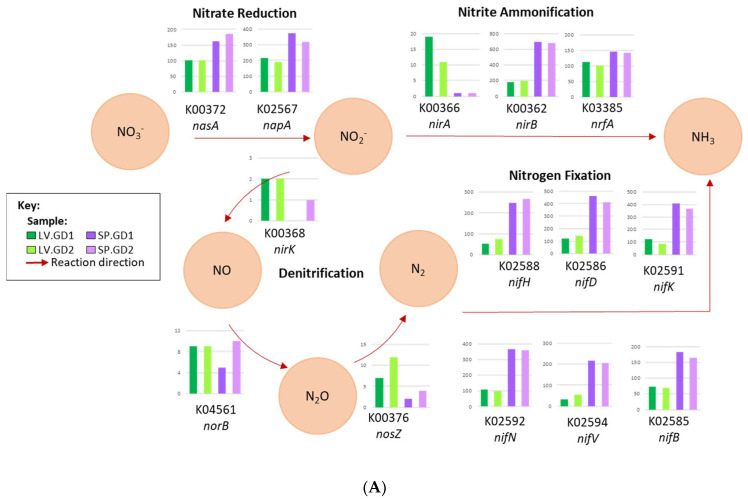

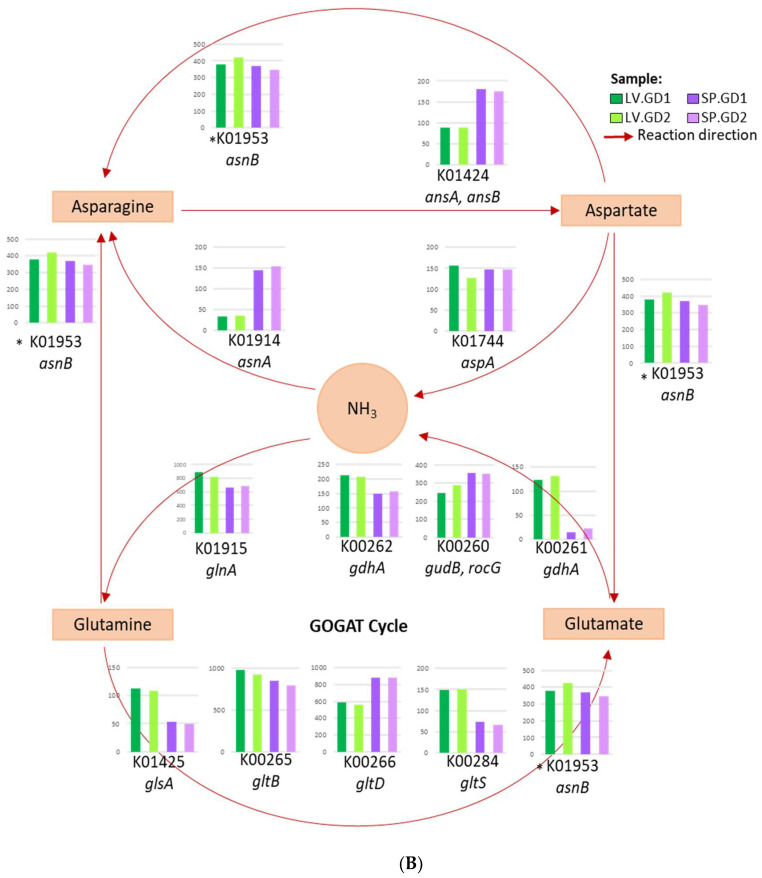
(**A**) The KEGG Orthology (KO) functional categories comprising the KEGG-Level-3 pathway of nitrogen metabolism (00910), including the direction of the reaction, were mapped. Inorganic nitrogen metabolism was mapped to show the substrate and product of each indicated reaction as it pertains to nitrate (NO_3_^−^) reduction, nitrite (NO_2_^−^) ammonification, denitrification to nitric oxide (NO), nitrous oxide (N_2_O), and nitrogen gas (N_2_), and nitrogen fixation into ammonia (NH_3_). (**B**) The KEGG Orthology (KO) functional categories comprising the KEGG-Level-3 pathway of nitrogen metabolism (00910), including the direction of the reaction, were mapped. The assimilation of ammonia into the amino acids glutamine and asparagine are also shown, including the subsequent enzyme-catalyzed amino acid transitions. The count data associated with the specified gene are shown as column bar graphs for each sample, including the KO category (KO number) and the gene name assigned through the KEGG database. Each category was color-coded according to their sample indicated in the color key as follows: SP.GD = purple sea urchin *Strongylocentrotus purpuratus* gut digesta; SP.GD1 = purple and SP.GD2 = light purple; LV.GD = green sea urchin *Lytechinus variegatus* gut digesta; LV.GD1 = green and LV.GD2 = light green. The metabolic pathways were elaborated using the information provided through KEGG Mapper as implemented in MG-RAST (v4.0.3). * The KEGG number K01953 corresponding to the *asnB* gene is listed multiple times due to its function in the generation of glutamate and asparagine from glutamine and aspartate.

An elaboration of the nitrogen metabolism pathway related to amino acid metabolism through KEGG Mapper showed pathways involved in ammonia (NH_3_) production to be abundant (Figure 5A,B). In the nitrate reduction pathway, the *nasA* assimilatory nitrate reductase catalytic subunit (KO number: K00372) and *napA* periplasmic nitrate reductase (KO number: K02567) was heightened, as well as nitrite reduction *nirB* (KO number: K00362) and *nrfA* (KO number: K03385) corresponding to nitrite reductase (NADH) large subunit and nitrite reductase (cytochrome c-552), respectively (Figure 5A). Nitrogen fixation was also represented by the *nif* cluster, which included the nitrogenase iron protein (nifH; KO number: K02588), nitrogenase molybdenum-iron protein alpha (nifD; KO number: K02586) and beta-chain (*nifK*; KO number: K02591), nitrogenase molybdenum-cofactor synthesis protein (*nifE*; KO number: K02587), nitrogen fixation protein (*nifB*; KO number: K02585), nitrogenase molybdenum-iron protein (*nifN*; KO number: K02592), and nitrogen fixation homocitrate synthase (*nifV*; KO number: K02594) at a noticeable abundance, particularly in the purple urchins. The genes in ammonia production that were noticeably abundant included glutamate dehydrogenase, and aspartate ammonia-lyase, which are elaborated as part of the ammonia assimilatory pathway. 

### 3.6. Genes for Ammonia Assimilation into Glutamine and Asparagine 

Genes involved in the assimilation of ammonia into various amino acids, namely, glutamine and asparagine, were observed as part of the nitrogen metabolism pathway (Figure 5B). For the assimilation of ammonia to generate glutamine, glutamine synthetase (*glnA*; KO number: K01915) was abundant in both the green and purple urchins. Similarly, genes involved in the transition of glutamine to glutamate were also heightened, which included glutamate synthase ferredoxin (*gltS*; KO number: K00284), glutaminase (*glsA*; KO number: K01425), and both the glutamate synthase (NADPH) large chain (*gltB*; KO number: K00265) and small chain (*gltD*; KO number: K00266); also included in this pathway was asparagine synthase (*asnB*; KO number: K01953), which hydrolyzes glutamine and aspartate to produce glutamate and asparagine. In the generation of ammonia from glutamate, glutamate dehydrogenase (*gudB*, rocG; KO number: K00260) and glutamate dehydrogenase (NADP+) (*gdhA*; KO numbers: K00261 and K00262) were abundant. For the assimilation of ammonia into asparagine, aspartate-ammonia ligase (*asnA*; KO number: K01914) was represented in both the green and purple urchins. Asparagine synthase (*asnB*; KO number: K01953) was also included in this pathway and denoted by an asterisk due to its dual function in generating both asparagine and glutamate from glutamine as indicated above. Additionally, L-asparaginase (*ansA* and *ansB*; KO number: K01424), which is involved in the synthesis of aspartate from asparagine, was represented in the continuation of this cycle. Last, aspartate ammonia-lyase (*aspA*; KO number: K01744) was also represented, generating ammonia and fumarate from L-aspartate. 

## 4. Discussion

The overall bacterial community composition determined from a single pooled DNA sample of green or purple sea urchin gut digesta at the phylum (e.g., Proteobacteria, Bacteroidetes, and Firmicutes) and the class levels (e.g., Gammaproteobacteria and Alphaproteobacteria) resulted from our study showed a similar trend in the gut of other Echinoderms such as sea cucumbers [62,63], sea stars [64], and brittle stars [65], as well as in a broad range of marine invertebrates such as sponges [66], tunicates [67], marine copepods [68], and corals [69]. In addition, previous studies in our laboratory also had similar microbial taxa in these sea urchin gut digesta based on high-throughput amplicon sequencing of the 16S rRNA gene [20,25]. Thus, both shotgun and 16S rRNA amplicon-based metagenomics approaches portrayed a commonality in bacterial taxa in Echinoderms and other marine invertebrates.

At the genus level, the high prevalence of the psychrophilic bacterium, *Psychromonas* (Gammaproteobacteria), within the purple urchin digesta could be due to the colder yearly water temperatures (10–12.8 °C; average = 11.7 °C) in the Oregon Coast compared to the Florida Coast (13.3–30 °C; average = 22 °C) (www.nodc.noaa.gov/dsdt/cwtg/npac.html (accessed on 27 May 2018)). In contrast, the presence of a high relative abundance of *Vibrio* in both sea urchins could be due to their tolerance to a wider range of temperatures [25,70,71]. However, whether the variation of water temperatures was the driving factor for the differences in the taxa abundances is speculative due to the limited sample size used in this study. Earlier studies have indicated *Vibrio* to be commonly found in sea urchins from diverse marine habitats [21,72,73,74]. Such ubiquity of *Vibrio* in sea urchins has been implicated in the metabolism of carbohydrate-rich algae and seagrass as their primary food source [21,72,73].

Similarly, the metabolic benefit of the *Psychromonas* in the purple urchin could also be carbohydrate-related metabolism and ω-3 polyunsaturated fatty acids (PUFA) production [17,75,76,77,78]. However, future experiments are to be conducted by culturing and subsequent biochemical tests to confirm the metabolic roles of these vibrios in our samples. In a recent study, the microbiota in the gut ecosystems of red urchin *Mesocentrotus franciscanus* and the purple urchin *Strongylocentrotus purpuratua* from Southern California Kelp Forest and barrens of Southern California revealed complex habitat-specific differences [79]. The taxa distribution in these urchins and our study mainly showed insignificant commonality. Such habitat-specific diverse taxa distribution in urchins has been implicated in the available diet, temperature, salinity, and other environmental factors [79]. Interestingly, the gut microbiota of red urchin *Loxechinus albus* from aquaculture environment [80], Antarctic heart urchins (Spatangoida) *Abatus agassizii* [81], green urchins *Lytechinus variegatus* from the U.S. Gulf of Mexico [24,25,82,83,84], and purple urchins *Strongylocentrotus purpuratus* from the U.S. Oregon Coast [20] showed abundant members of the family Campylobacteraceae. In contrast, *Strongylocentrotus purpuratus* from Southern California Kelp Forest and barrens had insignificant abundance (<1%) of the Campylobacteraceae [79]. This observation further emphasizes the possibility that diet in the inhabiting ecosystems likely promotes specific taxonomic composition in the urchin gut environment [85].

The use of shotgun metagenomic sequencing in this study revealed genes within the KEGG-Level-1 Orthology reference hierarchy of “metabolism” (K09100). Carbohydrate and amino acid metabolisms identified from KEGG-Level-2 indicated a high genetic potential of digestion and nutrient assimilation from the natural food source by the microbial communities in green and purple urchin digesta. These results support and expand our knowledge beyond previously predicted metabolic profiles determined by our laboratory in the gut digesta of both green and purple urchins by using Phylogenetic Investigation of Communities by Reconstruction of Unobserved States (PICRUSt) analysis [20,25]. 

All animals require fixed nitrogen, which is typically conducted by nitrogen-fixing bacteria to synthesize amino acids and nucleotides [86]. In particular, bacteria in the gut ecosystems of herbivores play a vital role in generating bioavailable forms of nitrogen by recycling nitrogen from organic molecules, reducing marine nitrate/nitrite, or fixing elemental nitrogen into ammonia [35]. Such metabolisms have been reported in diverse marine invertebrates [35], including bivalves [86,87,88] and sea urchins [89,90]. Similar to the results of our study, the nitrate/nitrite reduction pathways and nitrogen fixation have also been described in the marine sponge *Hymeniacidon heliophila*, emphasizing the assimilation into organic molecules [91]. Notably, previous studies on sea urchin gut bacteria have linked these microbial-mediated nitrogen metabolic processes benefitting the host and nutrient enrichment at the trophic levels. Specifically, bacteria of the sea urchin gut system have been suggested to play important roles in synthesizing essential amino acids [22]. Other studies have suggested nitrogenase-positive *Vibrio* isolates from the gut to provide a source of fixed nitrogen to the sea urchin host [89,90].

Moreover, studies performed on kelp-feeding *Strongylocentrotus droebachiensis* sea urchin aggregates have demonstrated an increase in the organic nitrogen fraction of their egesta at a rate of 0.21 g nitrogen m^2^/day. This activity indicates that the egesta are an energy-rich substrate to neighboring marine organisms at various trophic levels [15]. In our study, we obtained new insights into the genes involved in the nitrogen fixation and assimilatory reduction of nitrate and nitrite to ammonia in the sea urchin gut digesta compared to the studies reported by other investigators using non-genomic approaches [22,89,90]. Specifically, the genes involved in the assimilation pathway of ammonia to amino acids and nucleotides by the synthesis of glutamine and asparagine indicate that the gut digesta is likely enriched with essential nutrients. Although our study had limited metagenomic samples, the results indicate that the microbiota-driven metabolic pathway for the assimilation of excess ammonia may help to reduce the toxic effect to the sea urchins, and the inhibition of the nitrate and nitrite reduction pathway may further elucidate this metabolic pathway [92,93,94,95]. Moreover, the mucous-enveloped gut digesta formed shortly after the ingestion of food functions to maintain the necessary anaerobic environment for carbohydrate fermentation and the utilization of alternative inorganic electron acceptors in the nitrogen reduction pathways [23]. Future studies to elucidate the functionality of the metabolic genes require a larger sample size to better understand the role of the microbiota in the gut digesta to benefit the sea urchin nutrition during their passage through the gut lumen and impact trophic levels following egestion.

## Figures and Tables

**Table 1:** Sample statistics determined following high-throughput shotgun sequencing on the Illumina HiSeq platform, sequence processing, taxonomic, and functional assignments. The total sequences before and after host sequence removal are shown, and the following quality checking through MG-RAST (v4.0.3). The sequences assigned to domain Bacteria through RefSeq. High-quality sequences that did not receive a taxonomic identity were listed as “Other Seqs”. Alpha diversity was determined for each sample based on the Shannon and Simpson metrics as implemented through QIIME (v1.9.1) at the species level of taxonomic resolution. The number of sequences assigned to a functional gene through KEGG Orthology (KO) was also listed.

Sample	Total Seqs	Host-Removed Seqs	QC Seqs	Bacteria	Other Seqs	Shannon	Simpson	KO Functions
LV.GD1	11,179,611	1,903,485	1,879,183	646,758	3	8.99	0.996	206,021
LV.GD2	12,989,418	2,191,267	2,162,089	747,440	4	8.99	0.996	238,974
SP.GD1	11,280,351	6,232,967	6,082,068	3,323,484	4	7.47	0.965	1,103,493
SP.GD2	12,994,228	7,169,738	6,976,796	3,823,945	12	7.47	0.965	1,265,954

**Table 2:** Phylum (class for Proteobacteria) level heatmap table of the green and purple sea urchin gut digesta based on the taxonomic assignments determined through RefSeq. Phyla represented at <1% across all samples were listed as “Other”. The minimum and maximum relative abundance values were used to establish the gradient color scale, with 0.56% set at the minimum and 61.68% at the maximum. The gradient percentage values are indicated as follows: red = low; yellow = intermediate; green = high relative abundance. The table was generated using Microsoft Excel software (Microsoft, Seattle, WA, USA) using the conditional formatting option, and samples are indicated as follows: LV.GD = green sea urchin *Lytechinus variegatus* gut digesta; SP.GD = purple sea urchin *Strongylocentrotus purpuratus* gut digesta.

Phylum	LV.GD1	LV.GD2	SP.GD1	SP.GD2
Actinobacteria	2.99%	2.95%	0.69%	0.68%
Bacteroidetes	16.29%	16.21%	12.89%	12.84%
Cyanobacteria	2.60%	2.62%	0.62%	0.60%
Firmicutes	5.85%	5.85%	6.13%	6.10%
Fusobacteria	0.89%	0.87%	3.07%	3.13%
Planctomycetes	4.96%	5.02%	0.56%	0.56%
Proteobacteria (Alphaproteobacteria)	20.45%	20.61%	2.56%	2.55%
Proteobacteria (Betaproteobacteria)	3.17%	3.22%	1.60%	1.61%
Proteobacteria (Deltaproteobacteria)	4.65%	4.75%	5.61%	5.60%
Proteobacteria (Epsilonproteobacteria)	2.52%	2.50%	0.68%	0.66%
Proteobacteria (Gammaproteobacteria)	29.83%	29.55%	61.64%	61.68%
Verrucomicrobia	1.74%	1.78%	0.75%	0.75%
Other	4.06%	4.06%	3.19%	3.25%

## Data Availability

The pre-publication data access to the editor and the reviewers has been established through MG-RAST Token: https://www.mg-rast.org/mgmain.html?mgpage=token&token=SXin6UhaYUibLXwew5qITNgiBnLLfU7R1EDVKJtflNwMUwUcOi (accessed on 21 May 2021). We will make these data public access following acceptance of the manuscript for publication.

## Supplementary Materials

The Supplementary Materials are available online at https://www.mdpi.com/article/10.3390/cimb43020070/s1.
