# Supplementary material for: Microbial Composition and Genes for Key Metabolic Attributes in the Gut Digesta of Sea Urchins Lytechinus variegatus and Strongylocentrotus purpuratus Using Shotgun Metagenomics
Source: Curr Issues Mol Biol. 2021 Aug 26;43(2):978–95. doi: 10.3390/cimb43020070 (PMC8929034; doi:10.3390/cimb43020070)
Supplement: Supplementary file 1 [file cimb-43-00070-s001.zip › cimb-1328494-supplementary/Supplementary_Figure_S1_Hakim_et_al_CIMB.pdf]

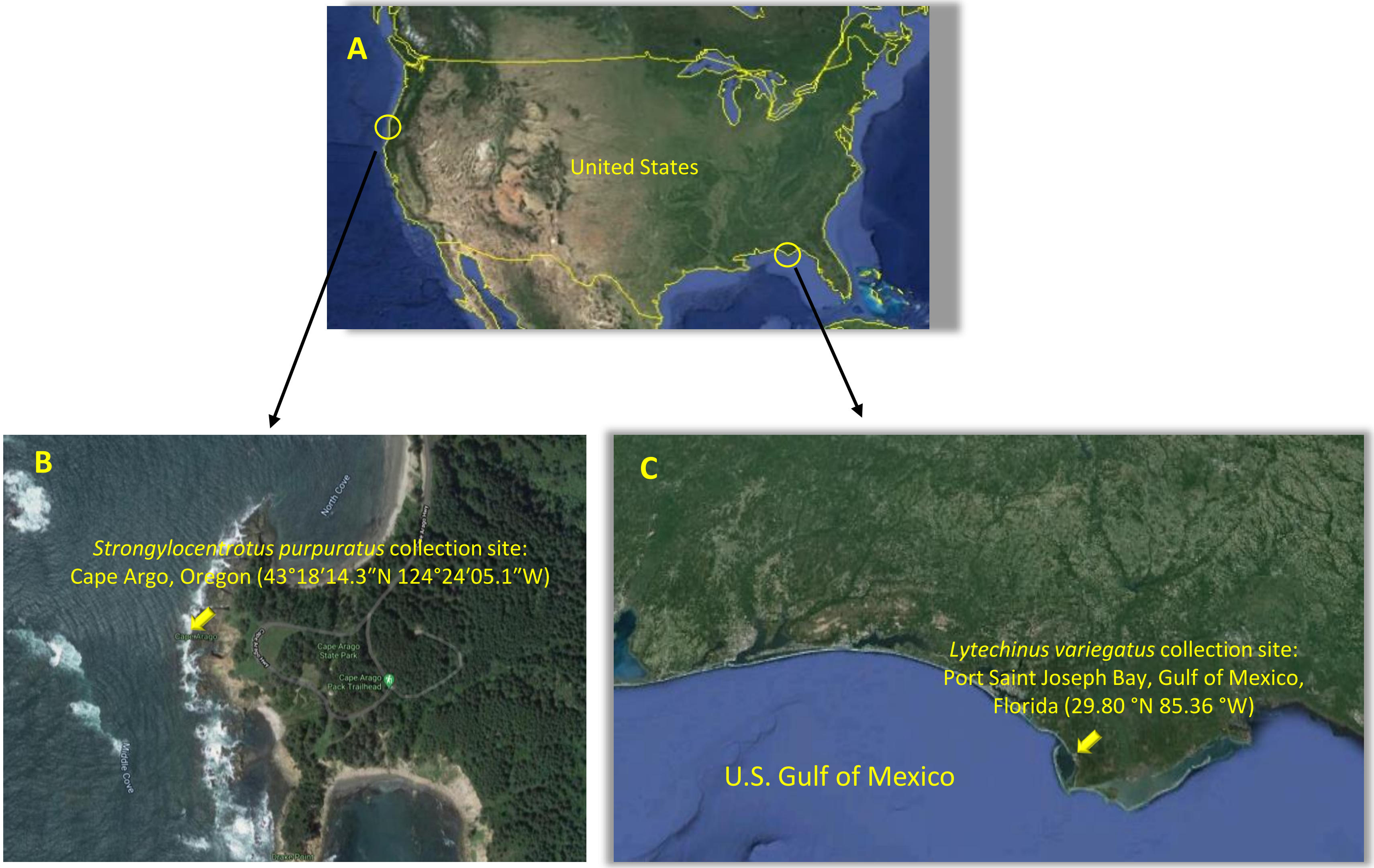

Supplementary Figure S1: Sample collection locations of green and purple urchins. **(A)** Satellite image of the collection site (circles) provided through Google Earth Pro (v.7.3.2.5491) (Data SIO, NOAA, US Navy NGA, GEBCO, Image Landsat/Copernicus; US Dept. of State Geographer; image date: December 2015); **(B)** The purple urchins (*S. purpuratus*) collection site at the Cape Argo, Oregon (43°18′14.3″N 124°24′05.1″W); **(C)** The green urchins (*L. variegatus*) collection site at the coastal waters at the Port Saint Joseph Bay Aquatic Preserve, Florida of the U.S. Gulf of Mexico (29.80 °N 85.36 °W).
