# Supplementary material for: Microbial Composition and Genes for Key Metabolic Attributes in the Gut Digesta of Sea Urchins Lytechinus variegatus and Strongylocentrotus purpuratus Using Shotgun Metagenomics
Source: Curr Issues Mol Biol. 2021 Aug 26;43(2):978–95. doi: 10.3390/cimb43020070 (PMC8929034; doi:10.3390/cimb43020070)
Supplement: Supplementary file 1 [file cimb-43-00070-s001.zip › cimb-1328494-supplementary/Supplementary_Figure_S2_Hakim_et_al_CIMB.pdf]

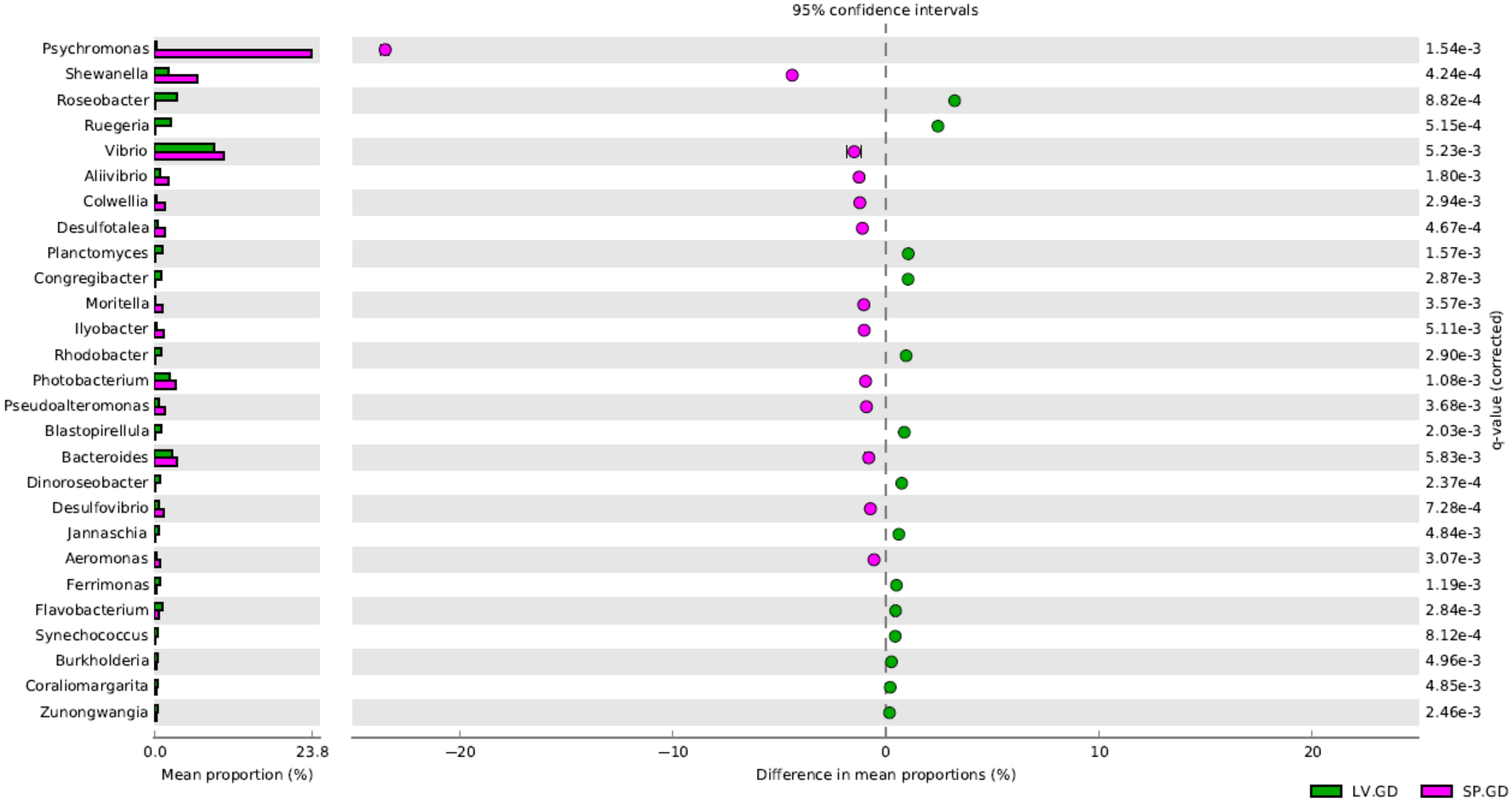

Supplementary Figure S2: Extended error bar analysis conducted on the top 50 genera determined in the sea urchin gut digesta. The analysis was performed using Statistical Analysis of Metagenomic Profiles (STAMP; v2.1.3) on the top 50 genera from the Bacteriome, determined through RefSeq as implemented in MG-RAST (v4.0.3). Technical replicates were grouped and normalized according to relative abundance. A two-sided Welch's t-test was performed between groups, and *p*-values were corrected using the Benjamini-Hochberg False Discovery Rate (FDR) correction (*q*-values). Genera showing significant differences between groups (*q*-value < 0.01) were listed, along with the relative abundance bar graphs (left) and the difference in mean proportions (center). Taxa identified as "Other" or "Unclassified" were excluded from the figure for better representation at the genus level. Groups are indicated as follows: LV.GD = green sea urchin *Lytechinus variegatus* gut digesta; SP.GD = purple sea urchin *Strongylocentrotus purpuratus* gut digesta. Taxonomic data generated through RefSeq as implemented in MG-RAST (v4.0.3).
