# Supplementary material for: Microbial Composition and Genes for Key Metabolic Attributes in the Gut Digesta of Sea Urchins Lytechinus variegatus and Strongylocentrotus purpuratus Using Shotgun Metagenomics
Source: Curr Issues Mol Biol. 2021 Aug 26;43(2):978–95. doi: 10.3390/cimb43020070 (PMC8929034; doi:10.3390/cimb43020070)
Supplement: Supplementary file 1 [file cimb-43-00070-s001.zip › cimb-1328494-supplementary/Supplementary_Table_S1_CIMB.pdf]

Supplementary Table S1: The relative abundances and two-group statistical results of the top 50 genera determined in the green and purple sea urchin gut digesta have been elaborated. Technical replicates were grouped and normalized by relative abundance. A two-sided Welch's t-test was performed between groups, and *p*-values were corrected using the Benjamini-Hochberg False Discovery Rate (FDR) correction (*q*-values). Taxa identified as "Other" and "Unclassified" are also indicated. Shown is the relative abundance per group including the standard deviation, the *p*-values, corrected *p*-values (*q*-values), and the difference between means (effect size). Groups are indicated as follows: LV.GD = green sea urchin *Lytechinus variegatus* gut digesta; SP.GD = purple sea urchin *Strongylocentrotus purpuratus* gut digesta. Taxonomic data generated through RefSeq as implemented in MG-RAST (v4.0.3).

Genus	LV.GD relative abundance (%)	LV.GD standard deviation (%)	SP.GD relative abundance (%)	SP.GD standard deviation (%)	*p*-values	*q*-values	effect size
Aeromonas	0.291	0.003	0.839	0.001	1.14E-03	3.07E-03	-0.548
Aliivibrio	0.875	0.000	2.121	0.002	5.31E-04	1.80E-03	-1.246
Bacillus	0.636	0.003	0.638	0.001	5.46E-01	5.46E-01	-0.002
Bacteroides	2.642	0.029	3.436	0.020	3.43E-03	5.83E-03	-0.794
Blastopirellula	0.950	0.000	0.078	0.001	6.36E-04	2.03E-03	0.873
Burkholderia	0.521	0.005	0.249	0.002	2.62E-03	4.96E-03	0.272
Campylobacter	0.685	0.013	0.107	0.003	1.10E-02	1.52E-02	0.578
Clostridium	1.512	0.006	1.880	0.014	9.98E-03	1.45E-02	-0.367
Colwellia	0.289	0.001	1.502	0.004	1.15E-03	2.94E-03	-1.213
Congregibacter	1.119	0.005	0.069	0.001	1.01E-03	2.87E-03	1.050
Coraliomargarita	0.569	0.000	0.357	0.001	2.66E-03	4.85E-03	0.212
Cytophaga	0.553	0.016	0.281	0.002	3.46E-02	3.76E-02	0.271
Desulfotalea	0.528	0.002	1.620	0.002	9.16E-06	4.67E-04	-1.091
Desulfovibrio	0.687	0.004	1.405	0.005	1.14E-04	7.28E-04	-0.718
Dinoroseobacter	0.793	0.001	0.041	0.001	9.28E-06	2.37E-04	0.752
Ferrimonas	0.780	0.005	0.276	0.005	2.11E-04	1.19E-03	0.504
Flavobacterium	1.156	0.011	0.691	0.010	1.17E-03	2.84E-03	0.465

Fusobacterium	0.409	0.003	1.406	0.019	9.50E-03	1.42E-02	-0.997
Geobacter	0.499	0.010	0.375	0.004	2.98E-02	3.38E-02	0.124
Gramella	0.918	0.017	0.442	0.002	2.17E-02	2.63E-02	0.476
Ilyobacter	0.331	0.005	1.342	0.013	2.61E-03	5.11E-03	-1.010
Jannaschia	0.669	0.003	0.053	0.001	2.37E-03	4.84E-03	0.616
Maribacter	0.626	0.009	0.344	0.000	2.12E-02	2.64E-02	0.282
Marinomonas	0.272	0.001	0.748	0.012	1.60E-02	2.03E-02	-0.476
Marivirga	0.494	0.005	0.211	0.000	1.16E-02	1.55E-02	0.283
Moritella	0.100	0.001	1.126	0.005	1.68E-03	3.57E-03	-1.026
Paludibacter	0.400	0.001	0.564	0.005	1.38E-02	1.80E-02	-0.163
Parabacteroides	0.598	0.014	0.782	0.004	3.43E-02	3.80E-02	-0.184
Pedobacter	0.555	0.007	0.498	0.001	6.72E-02	6.85E-02	0.057
Photobacterium	2.348	0.009	3.294	0.010	2.12E-04	1.08E-03	-0.945
Pirellula	0.900	0.015	0.040	0.001	1.08E-02	1.53E-02	0.859
Planctomyces	1.216	0.003	0.160	0.001	4.30E-04	1.57E-03	1.057
Polaribacter	0.602	0.012	0.322	0.001	2.45E-02	2.90E-02	0.279
Porphyromonas	0.361	0.004	0.471	0.009	2.50E-02	2.90E-02	-0.110
Prevotella	0.643	0.019	0.838	0.007	3.81E-02	4.05E-02	-0.196
Pseudoalteromonas	0.623	0.000	1.526	0.003	1.66E-03	3.68E-03	-0.904
Pseudomonas	1.062	0.006	0.722	0.026	3.90E-02	4.06E-02	0.340
Psychromonas	0.314	0.001	23.790	0.016	3.93E-04	1.54E-03	-23.476
Rhodobacter	1.032	0.005	0.073	0.001	1.25E-03	2.90E-03	0.959
Rhodopirellula	1.791	0.017	0.265	0.000	6.96E-03	1.14E-02	1.526
Roseobacter	3.390	0.001	0.156	0.003	1.04E-04	8.82E-04	3.234
Roseovarius	0.721	0.008	0.052	0.000	7.33E-03	1.17E-02	0.669
Ruegeria	2.569	0.006	0.118	0.004	3.03E-05	5.15E-04	2.451
Shewanella	2.083	0.011	6.470	0.015	4.16E-05	4.24E-04	-4.386
Synechococcus	0.563	0.003	0.115	0.002	1.12E-04	8.12E-04	0.448
Vibrio	9.043	0.050	10.519	0.058	2.98E-03	5.23E-03	-1.476
Zunongwangia	0.425	0.002	0.245	0.003	8.21E-04	2.46E-03	0.179

unclassified (Flavobacteriales)	0.478	0.005	0.211	0.001	8.11E-03	1.25E-02	0.266
unclassified (Gammaproteobacteria)	2.051	0.010	0.276	0.005	3.86E-04	1.64E-03	1.775
unclassified (Rhodobacteraceae)	0.638	0.002	0.035	0.001	2.48E-04	1.15E-03	0.602
Other	46.692	0.080	26.820	0.068	3.55E-05	4.53E-04	19.872
